# Maternal care of heterozygous dopamine receptor D4 knockout mice: Differential susceptibility to early‐life rearing conditions

**DOI:** 10.1111/gbb.12655

**Published:** 2020-06-15

**Authors:** Jelle Knop, Marinus H. van IJzendoorn, Marian J. Bakermans‐Kranenburg, Marian Joëls, Rixt van der Veen

**Affiliations:** ^1^ Department of Translational Neuroscience, Brain Center Rudolf Magnus University Medical Center Utrecht, Utrecht University Utrecht The Netherlands; ^2^ Faculty of Social and Behavioural Sciences Leiden University Leiden The Netherlands; ^3^ Department of Psychology, Education and Child Studies Erasmus University Rotterdam Rotterdam The Netherlands; ^4^ Primary Care Unit, School of Clinical Medicine University of Cambridge Cambridge UK; ^5^ Clinical Child & Family Studies Vrije Universiteit Amsterdam Amsterdam The Netherlands; ^6^ University of Groningen, University Medical Center Groningen Groningen The Netherlands

**Keywords:** animal model, communal nesting, differential susceptibility, dopamine receptor D4, early‐life adversity, gene‐environment interaction, intergenerational transmission, limited bedding/nesting, maternal care, puberty onset

## Abstract

The differential susceptibility hypothesis proposes that individuals who are more susceptible to the negative effects of adverse rearing conditions may also benefit more from enriched environments. Evidence derived from human experiments suggests the lower efficacy dopamine receptor D4 (*DRD4*) 7‐repeat as a main factor in exhibiting these for better and for worse characteristics. However, human studies lack the genetic and environmental control offered by animal experiments, complicating assessment of causal relations. To study differential susceptibility in an animal model, we exposed *Drd4* ^*+/−*^ mice and control litter mates to a limited nesting/bedding (LN), standard nesting (SN) or communal nesting (CN) rearing environment from postnatal day (P) 2‐14. Puberty onset was examined from P24 to P36 and adult females were assessed on maternal care towards their own offspring. In both males and females, LN reared mice showed a delay in puberty onset that was partly mediated by a reduction in body weight at weaning, irrespective of *Drd4* genotype. During adulthood, LN reared females exhibited characteristics of poor maternal care, whereas dams reared in CN environments showed lower rates of unpredictability towards their own offspring. Differential susceptibility was observed only for licking/grooming levels of female offspring towards their litter; LN reared *Drd4* ^*+/−*^ mice exhibited the lowest and CN reared *Drd4* ^*+/−*^ mice the highest levels of licking/grooming. These results indicate that both genetic and early‐environmental factors play an important role in shaping maternal care of the offspring for better and for worse.

## Graphical abstract

Heterozygous dopamine receptor D4 knock‐out mice (Drd4^/−^) reared in different environments exhibited alterations in licking/grooming behavior towards their own offspring in line with the differential susceptibility theory.

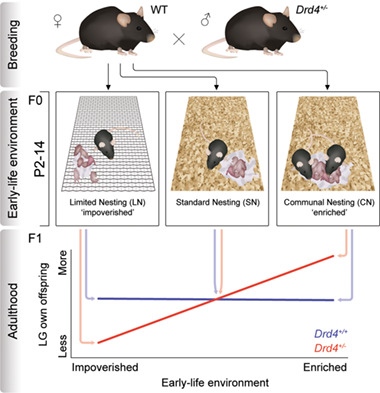

## 1 INTRODUCTION

### 1.1 Differential susceptibility

Parental care is essential for survival and development of newborn mammals, including humans. Variations in parental care substantially contribute to the environmental variability experienced by offspring. Extensive evidence indicates that poor parental care can contribute to increased vulnerability to develop later‐life psychopathology in humans and impaired cognitive performance in rodents.^1^, ^2^ This vulnerability crucially depends on a complex cross‐talk between an individual's genetic makeup and rearing environment.^3^ While the genetic background of some individuals is related to a vulnerable phenotype in the face of early‐life adversity, others appear to be more resilient. Interestingly, individuals who are genetically more susceptible to the detrimental consequences of negative (rearing) conditions may also experience greater benefits from a positive and stimulating (rearing) environment.^4^, ^5^ This crossover effect *for better and for worse*, also called differential susceptibility, is supported by studies investigating the role of human allelic variation across a variety of susceptibility genes.^6^

An example of such differentially susceptibility concerns the exon III 7‐repeat polymorphism of the D2‐like dopamine receptor D4 gene (*DRD4‐7R*). In humans, this variant has been associated with reduced gene expression and efficiency^7^, ^8^ and acts as a susceptibility marker of dopamine‐related genes.^6^ Carriers of this variant have an increased risk of developing externalizing problems in relation to parental insensitivity^9^ and chronic stress.^10^ However, these individuals also benefitted most from enhanced positive parenting.^11^ Meta‐analytic evidence further supports an important role of dopamine‐related genes in moderating susceptibility to both positive and negative rearing environments.^12^ Of note, the DRD4 also plays a role in moderating parental care itself.^13^, ^14^

### 1.2 Rodent models of impoverished or enriched rearing environments

Studying differential susceptibility in humans is hampered by random genetic variability. Moreover, it is often difficult to randomly allocate individuals to specific environments while also taking genotype into account. Therefore, we set out to study the causal contribution of decreased *Drd4* functioning to differential susceptibility with a truly randomized experiment in rodents, allowing strict control for both genetic variation and environmental factors.^15^ By using heterozygous dopamine receptor D4 knock‐out (*Drd4* ^*+/−*^) mice, we aimed to mimic the reduced *DRD4* efficiency observed in human *DRD4‐7R* allele carriers.

We selected two rodent models developed to chronically induce alterations in the quality and quantity of parental care received by offspring. First, limited availability of nesting and bedding (LN) material to a mouse dam was used to induce an adverse early life environment; this model increases unpredictability of maternal care received by the pups,^16^, ^17^, ^18^ leading to increased corticosterone levels in pups^19^ and altered offspring development and behavior in adulthood.^20^, ^21^ Second, as beneficial and stimulating social rearing environment we selected a communal nesting (CN) condition, where two or more dams share care‐giving behavior towards multiple litters.^22^ In this condition, pups experience higher levels of nest occupancy by at least one dam^18^, ^23^ and can interact with peers as well as siblings. Mice reared in communal nesting conditions exhibit various neurobiological and behavioral characteristics that are indicative of improved social competences.^24^

### 1.3 Outcome parameters

In line with a previous study,^18^ we focused on timing of puberty onset, a key moment in development that is malleable by environmental influences as part of an adaptive reproductive strategy.^25^ Although adverse rearing conditions in females are linked to accelerated pubertal onset in humans^26^ and rats,^27^ such effects have not yet been observed in mice.^18^, ^28^ In human males, adverse rearing conditions had no effect on puberty onset,^29^ while puberty onset in male rodents was either unaffected or delayed.^18^, ^27^, ^30^ However, rodent models of early‐life adversity (ELA) invariably decrease body weight gain, which is an important mediator of puberty onset. Therefore, it is unclear whether the delayed puberty onset observed in ELA reared animals is the result of decreased body weight gain or whether a *relative* acceleration irrespective of body weight exists in rodents as well.

A second outcome was maternal care provided by female offspring. In addition to sexual maturation, the theory submitted by Belsky et al^25^ predicted that variations in early parental care would have the potential to alter adult parental care in humans. Preclinical rodent studies allow for feasible, controlled intergenerational studies on maternal care and, in line with the life history theory, extensive evidence suggests that alterations in maternal care may be transmitted across generations.^31^ Variations in levels of licking/grooming (LG) behavior and arched‐back nursing (ABN), core features of positive parenting in rodents, have been shown to affect corticosterone reactivity, hippocampal development and maternal care of the offspring.^31^ In addition, the limited bedding/nesting model, which evokes changes in maternal care, results in aberrant patterns of maternal care of the offspring,^32^ whereas mice reared in a communal nesting condition display improved maternal behavior towards their own pups.^33^ Taken together, these studies highlight the importance of maternal care for offspring development, as well as the potential of maternal care to be shaped by the early‐life environment, contributing to the intergenerational transmission of social behavior.

In this study, we tested heterozygous *Drd4* knock‐out (*Drd4* ^*+/−*^) mice and control litter mates on susceptibility to both adverse (LN) and enriched (CN) rearing environments to model differential susceptibility in mice. Animals were examined on (a) puberty onset, to track early development, (b) their own maternal care towards the next generation as an indicator of transgenerational effects and (c) basal corticosterone levels, to investigate involvement of the hypothalamic‐pituitary‐adrenal‐axis (HPA‐axis) in differential susceptibility. Although puberty onset would be hypothesized to be accelerated in LN and delayed in CN reared animals according to life history theory, previous findings indicate that the opposite may be true in mice due to the strong effects of body weight. LN reared mice were hypothesized to display poor maternal care, whereas CN reared mice were hypothesized to show enhanced maternal care. To confirm differential susceptibility, these effects would have to be amplified in, or exclusive to, *Drd4* ^*+/−*^ mice.

## 2 MATERIALS AND METHODS

### 2.1 Animals and housing

**FIGURE 1:**
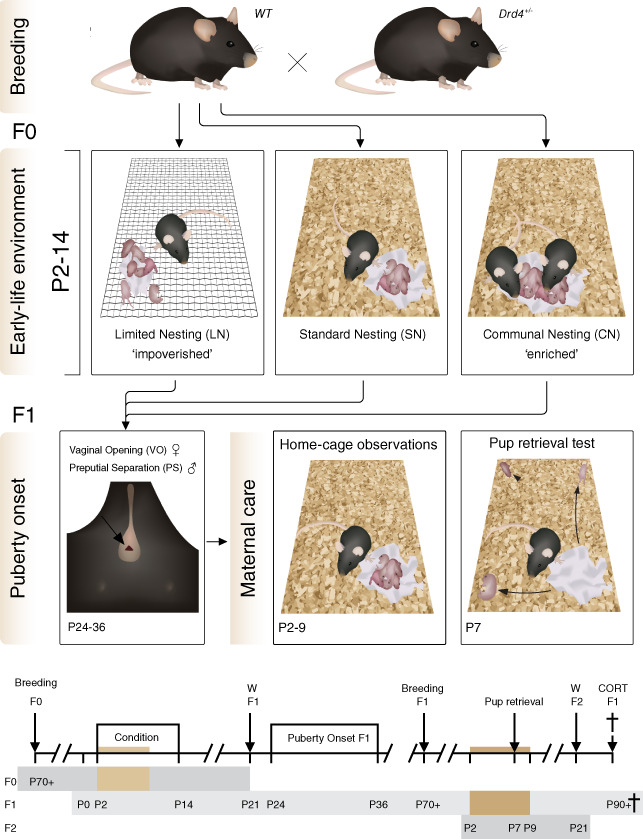
Outline of the experiments. Study design and timeline of the experiment. A wild‐type female was paired with a DRD4+/− male to obtain litters of mixed genetic background. Experimental time points for each generation of mice are depicted. W = weaning. P = postnatal day. Colored bars indicate periods of home cage maternal care observations

B6.129P2‐*Drd4* ^*tm1Dkg*^/J (*Drd4* ^*+/−*^) mice^34^ were originally obtained from the Jackson Laboratory (Bar Harbor, Maine, USA) and bred in‐house with C57BL/6JOlaHsd (breeding colony, originally obtained from Harlan, France) mice for at least four generations before experiments started. All breeding was performed in our own animal facility. Wild‐type (wt) female C57BL/6 mice were allowed to breed with male *Drd4* ^*+/−*^ mice to generate *Drd4* ^*+/−*^ F1 offspring and *Drd4* ^*+/+*^ control litter mates. *Drd4* ^*+/−*^ mice are viable, healthy and visually indistinguishable from control animals. Between postnatal day 2 and 14 (P2‐14), dam and litter were exposed to a limited nesting/bedding (LN), standard (SN) or communal nesting (CN) condition. A total of 129 female and 116 male F1 offspring obtained from 40 breedings was used to assess puberty onset and, in females (n = 75), maternal care of this generation (see Figure 1. for a timeline of the experiment). Final numbers per experimental group are depicted in figure legends and specified per litter in Table S1. Puberty onset and F1 maternal care were scored by a trained experimenter blind to rearing condition and genotype of the animals. Mice had ad libitum access to water and chow and were housed on a reversed LD cycle (lights off 08:00 am, temperature 21‐22°C, humidity 40%‐60%). All experiments were performed in accordance with the EC council directive (86/609/EEC) and approved by the Central Authority for Scientific Procedures on Animals in the Netherlands (CCD approval AVD115002016644).

### 2.2 Breeding conditions

Breeding was performed as described earlier.^18^ In short, one male was paired with two females for 4 days, after which females were co‐housed until approximately 1 week prior to birth. Pregnant dams were then housed in a type II short Macrolon cage (21.5 × 16 cm) with filter top and a Nestlet (5 × 5 cm, Technilab‐BMI, Someren, The Netherlands) as nesting material. Nestlets are made from sterilized cotton fiber material that the dam can use to shred and form a nest site while still allowing for observation of maternal behavior. Daily inspection for the birth of litters was conducted at 09:00 am, assigning the day prior as P0. At P2, dam and litters were weighed and randomly assigned to the LN, SN or CN condition. All litters were culled (or cross‐fostered if necessary) to six to seven pups per litter, with a maximum addition of one pup per litter and a minimum of two pups of each sex in each litter.

The LN condition consisted of placing the dam and litter in a cage with limited bedding material, made inaccessible by a stainless steel wired mesh. In addition only half the regular amount of nesting material (Nestlet, 5 × 2.5 cm) was available. In the SN condition, standard amounts of bedding (±3 cm bedding) and nesting material (Nestlet, 5 × 5 cm) were available to the dam. The CN paradigm consisted of co‐housing the experimental weight dam (and her genetically heterogeneous F1 litter) with another ear‐punched dam (and wt litter) in a type II regular Macrolon cage (32 × 16 cm, 5 × 5 cm Nestlet and regular bedding). The pups of this second mother were marked with surgical marker at P2 and P7 (ArcRoyal, Ireland) to ensure correct allocation of the pups to their mother at the end of communal housing at P14. At P9 and P14, all dams and litters were weighed and provided with clean cages, adding a bit of used bedding material to maintain odor cues. From P14 until weaning at P21, animals were housed in standard nesting conditions. All mice were weighed at weaning and ear punches were obtained to facilitate individual recognition and genotype offspring.

### 2.3 Maternal care observations F0

An instantaneous sampling method^18^ was used to score maternal behavior of the dams in different conditions. Three 75‐minute scoring sessions were performed daily from P2‐9 between 6:00‐7:30 am (end light phase), 12:00‐4:00 am (mid dark phase) and 6:30‐8:30 pm (end dark phase). Red light conditions were used to score during the dark phase sessions. Maternal behavior of each dam was scored every 3 minutes, leading to 25 observations per session and 75 observations per day for each dam. Maternal behaviors were classified as: arched‐back nursing (ABN), passive nursing, licking/grooming pups (LG), nest building, self‐grooming on nest, feeding and self‐grooming off nest. For observations during which the behavior did not qualify for one of these categories, only on or off nest location of the dam was scored. A Samsung Galaxy Note 4 with Pocket Observer 3.3 software (Noldus, the Netherlands) was used for behavioral scoring, and data was analyzed using Observer XT 10.5 (Noldus, the Netherlands). Both dams in the communal nesting condition were scored separately, using average scores of each pair of dams as an indication of maternal behavior received by the litter.

Assessment of maternal care was performed by looking at (a) frequencies of the various maternal behaviors, (b) unpredictability of maternal care and (c) fragmentation, using on/off nest transitions. First, percentage of time spent on the various maternal behaviors was calculated per day (pooling the three daily sessions) or circadian phase (pooling over six postnatal days) to assess the development over days and circadian rhythmicity of maternal care, respectively. Second, overall unpredictability of maternal behavior was evaluated using the entropy rate of transitions between different maternal behaviors.^16^ The entropy rate is obtained by calculating the probabilities of certain maternal behaviors predicting specific subsequent behaviors, in which higher entropy rates are indicative of higher unpredictability. In addition, unpredictability of maternal care specifically on the nest site was calculated by pooling all off‐nest behaviors to enhance representation of the unpredictability rate as experienced by the offspring. Third, the average number of transitions from and to the nest site per observation was used as an index of fragmentation of maternal care.^19^

### 2.4 Puberty onset F1

As an external measure of puberty onset in males, mice were restrained and gently examined daily from P27 to P33 (10:00‐12:00 am) on the potential to fully retract the prepuce and expose the glans penis which was designated as puberty onset.^35^ Female mice were scored daily from P24 to P36 for vaginal opening, here taken as sign of puberty onset.^36^ All mice were weighed at puberty onset.

### 2.5 Maternal care F1

During adulthood (>P70), female F1 mice were allowed to breed with a wild‐type male as described for F0. All F2 litters were culled/cross‐fostered to six pups and reared in standard nesting conditions. At P2, P9, P14 and P21, clean cages were provided and animals were weighed. Maternal care observations were performed as described for F0 maternal behavior. At P7 between 10:00‐12:00 am, pup retrieval behavior was measured using a 5 minute pup retrieval test as described earlier.^18^ If a dam did not retrieve all three pups within 5 minutes, a latency of 300 seconds was assigned.

### 2.6 Plasma corticosterone levels F1

To measure plasma corticosterone levels, all F1 dams were decapitated in random order between 1:00 and 5:00 pm at least 3 weeks after weaning of F2 litters. Trunk blood was collected in heparin containing tubes (Sarstedt, The Netherlands) on ice and centrifuged for 10 minutes (15 682 rcf) at 4°C. Plasma was collected and stored at −20°C until radioimmunoassay (MP Biomedicals, The Netherlands; sensitivity 3 ng/mL).

### 2.7 Statistical analysis

Data are expressed as mean ± sem. Values deviating >3.29 sd from the mean were defined as outlying and winsorized accordingly.^37^ The entropy rate of one F0 LN dam was winsorized. Data were analyzed using SPSS 23 (IBM) and litter effects in all F1 measures were accounted for using the SPSS complex samples module. However, no effect sizes are provided in this model. In other analyses, eta squared effect sizes (*η* ^*2*^), representing the explained variance relative to the total model variance, are reported. Overall anova statistics are presented in Tables S1‐S3, Tukey hsd (main effects) or Sidak (interactions) corrected post hoc comparisons are depicted in figures.

Greenhouse‐Geisser corrected repeated measures anovas with breeding condition as the between‐subject factor and postnatal day or observation as within‐subject factors were used to analyze F0 maternal behaviors. Maternal behaviors from two observation sessions at P2 were analyzed separately to dissociate acute effects of novel environment exposure from more chronic alterations in maternal care. P2 maternal behavior, entropy rates and fragmentation were analyzed using a one‐way anova with breeding condition as the between‐subjects factor. Pup retrieval latencies of F1 dams were analyzed using cox regression, as this method is preferred if a subset of animals fails to complete a certain task.^38^ All other F1 measures were analyzed using a two‐way anova including rearing condition and genotype as independent variables. Pearson correlations were used for correlational data. Mediation analysis was conducted using the PROCESS v3 SPSS macro,^39^ with rearing condition as a multicategorical independent variable and the SN group as the reference category. The day of puberty onset was used as dependent variable and body weight at weaning and received entropy rates as potential mediators. Significant mediation was assigned when 95% confidence intervals of mediation did not include zero.

## 3 RESULTS

### 3.1 Maternal care by F0: care provided in an enriched or impoverished environment

**FIGURE 2:**
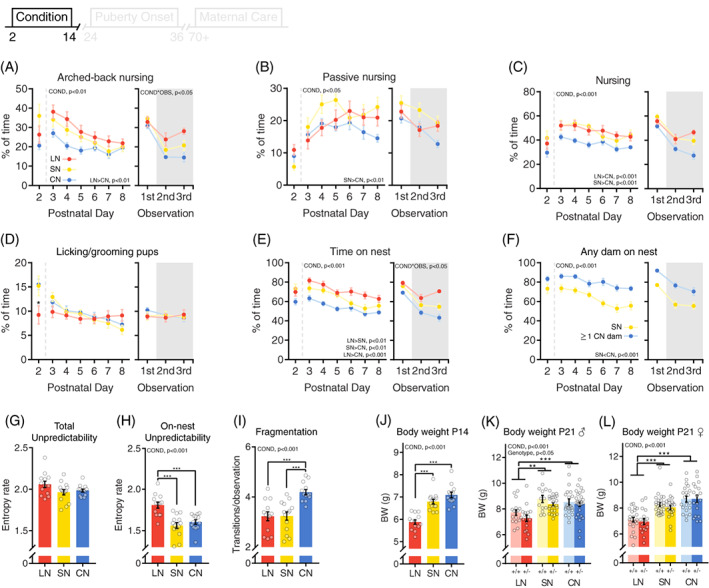
Effect of different housing conditions on F0 maternal care and F1 body weight. (A), Arched‐back nursing, (B) passive nursing, (C) total nursing, (D) licking/grooming and (E and F) time on nest for limited nesting (red, n = 13), standard nesting (yellow, n = 14) and communal nesting (blue, n = 13) dams, depicted over postnatal days (left) and time of the day (right). The shaded area indicates the dark phase of the LD cycle. Data in F represents the time on nest by at least one dam from the litters perspective. (G) Unpredictability of all scored maternal behaviors and (H) unpredictability of maternal care when all off‐nest behaviors were combined into one measure. (I) Fragmentation (on/off nest transitions) of maternal behavior. Each dot represents one dam and the average of two dams in the CN condition. (J) Offspring body weight averaged per litter at postnatal day 14. (K) Offspring body weight per individual at weaning for males and (L) females. +/+: control, +/−: heterozygous *Drd4*. Group size: ♂: LN +/+: n = 17, LN +/−: n = 16, SN +/+: n = 13, SN +/−: n = 23, CN +/+: n = 22, CN +/−: n = 27; ♀: LN +/+: n = 22, LN +/−: n = 17, SN +/+: n = 26, SN +/−: n = 22, CN +/+: n = 20, CN +/−: n = 18. ANOVA main effects are depicted in the top left of each figure. Post hoc comparisons are depicted bottom right or by lines. COND = main effect of condition. COND*OBS = condition*observation interaction effect. Asterisks indicate interactions or post‐hoc comparisons. *P < .05, **P < .01, ***P < .001

The maternal care of mouse dams was affected by environmental condition (Figure 2, Table S1). Arched‐back nursing (ABN) levels in LN dams were increased compared to CN dams (Figure 2A), while passive nursing was decreased in CN dams compared to SN dams (Figure 2B). Taking the sum of ABN and passive nursing together, total nursing levels displayed by individual CN dams were decreased compared to LN and SN dams (Figure 2C), but feeding behavior in the CN condition increased (Figure S1A). Although environmental conditions did not affect licking/grooming behavior from P3‐8, LG levels were affected more acutely at P2 (Figure 2D). Post hoc testing indicated that specifically pups in a LN setting were deprived from LG on this first day of novel environment exposure. Overall nest occupancy of LN dams was increased compared to SN and CN dams (Figure 2E), but this was mostly due to an increase in the time LN dams were engaging in non‐pup directed behaviors on the nest site (self‐grooming and other behavior, see Figure S1).

Despite a reduction of nest occupancy by individual CN dams compared to both LN and SN mice, the nest site in the CN setting had higher levels of nest occupancy by at least one dam compared to the SN condition (Figure 2F). Moreover, circadian rhythmicity of maternal behavior was altered by exposure to different conditions (Figure 2E, right panel). The pattern of maternal care displayed towards the end of the dark phase (third observation time‐point) was more comparable to the light phase (first observation time‐point) in LN dams, whereas CN and SN dams displayed similar levels of maternal behaviors during both dark phase observations (second and third observation time‐points). This pattern appeared to be consistent across different behaviors but reached significance for ABN, nest occupancy and off‐nest behaviors.

The overall unpredictability of behavior displayed by dams was not significantly affected by environmental condition (Figure 2G). However, unpredictability of behavior specifically on the nest site (on nest entropy rates) was altered (Figure 2H). Post hoc comparisons revealed that the LN dams displayed increased unpredictability of maternal care compared to the SN and CN dams. Nesting condition also affected fragmentation of maternal care, measured by the number of transitions from and to the nest site (Figure 2I); CN dams exhibited increased fragmentation compared to SN and LN dams.

### 3.2 Effects of enriched or impoverished rearing conditions on F1

#### 3.2.1 Effects of rearing conditions on early development

**FIGURE 3:**
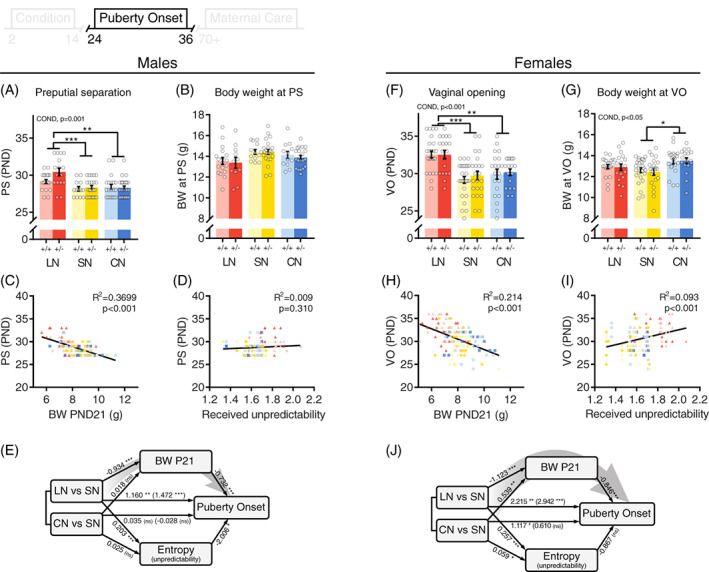
Effects of different rearing conditions on sexual maturation in male and female offspring. (A and F), Puberty onset in male (preputial separation) and female (vaginal opening) mice. (B and G), Body weight at puberty onset. (C and H), Body weight at weaning negatively correlated with puberty onset in both males and females, whereas (D and I) received on‐nest unpredictability rates during rearing positively correlated with puberty onset only in females. (E and J), Graphical representation of mediation models. Numbers represent estimated model coefficients, direct effects are depicted in parenthesis. Gray arrows indicate a significant mediation pathway. +/+: control, +/−: heterozygous *Drd4*. Asterisks indicate post hoc comparisons. *P < .05, **P < .01, ***P < .001

At P14, body weight of LN litters was decreased compared to SN and CN litters (Figure 2J), an effect that remained at weaning in both males (Figure 2K) and females (Figure 2l). Puberty onset was also affected by rearing condition in both males (Figure 3A) and females (Figure 3F); LN reared animals displayed a delay in puberty onset compared to SN and CN reared mice. In females (Figure 3G), but not males (Figure 3B), body weight at puberty onset was increased in CN reared animals compared to SN and LN mice. In both males and females, body weight at weaning negatively correlated with puberty onset (Figure 3C,H), whereas a positive correlation between received entropy rates during early development and puberty onset was only observed in females (Figure 3D,I). Mediation analysis revealed that in males, the delayed puberty onset observed in LN reared mice was partly mediated by the reduced body weight at weaning (95%CI = [0.36, 1.17], Figure 3E). In females, body weight at weaning was a significant mediator of puberty onset for both LN (95%CI = [0.36, 1.66], Figure 3J) and CN reared animals (95%CI = [−0.96, −0.08]). However, entropy rates did not mediate the effects of rearing condition on puberty onset (LN: 95%CI = [−1.21, 0.83]; CN: 95%CI = [−0.29, 0.23]).

#### 3.2.2 Maternal care by F1: effects of rearing conditions on later‐life maternal care

**FIGURE 4:**
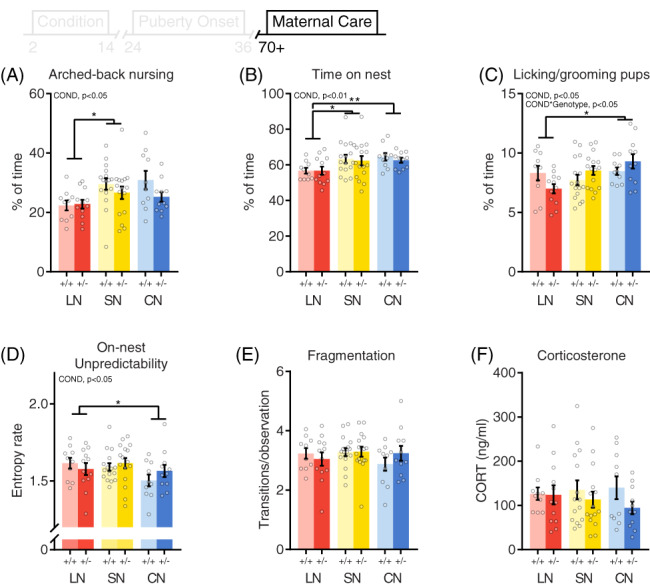
Effects of different rearing conditions and Drd4 genotype on maternal care and basal corticosterone levels in female F1 offspring. Overall (P2‐9) levels of (A) Arched‐back nursing, (B) time on nest and (C) licking grooming exhibited by F1 female dams. (D), On‐nest unpredictability and (E) fragmentation (on/off nest transitions) of maternal behavior. (F), Basal corticosterone levels. +/+: control, +/−: heterozygous *Drd4*. Group size: LN +/+: n = 10, LN +/−: n = 12, SN +/+: n = 16, SN +/−: n = 16, CN +/+: n = 10, CN +/−: n = 11). Asterisks indicate post hoc comparisons. *P < .05, **P < .01

Mice that were exposed to LN rearing conditions during early development displayed decreased levels of arched‐back nursing (ABN) towards their own offspring compared to SN‐reared animals (Figure 4A). While passive nursing levels were not affected by rearing condition (Figure S2A), total nursing behavior was decreased in LN reared mice compared to CN reared animals (Figure S2B). In addition, the total time spent on the nest site was decreased in LN‐reared animals compared to both SN and CN reared mice (Figure 4B). A main effect of rearing condition was also observed for the percentage of time dams spent licking/grooming their own pups, a key maternal behavior; LN‐reared dams spent less time licking/grooming than dams reared in a communal nesting environment (Figure 4c).

While F0 dams did not differ in total entropy rate, the total entropy rate of F1 maternal behavior was decreased in CN reared mice compared to dams reared in a SN environment (Figure S2C). In addition, CN‐reared dams displayed lower on‐nest unpredictability rates compared to LN reared animals (Figure 4C). Fragmentation of maternal care was not affected by early life condition. Thus, while CN animals were raised with more fragmented maternal care, they did not differ in this behavior themselves when allowed to breed in a standard nesting condition. Cox regression revealed that pup retrieval was unaffected by rearing condition (hazard ratio 95%CI = [0.72, 1.39], *P* = .986). Although P2 body weight of the next generation (F2) was decreased in offspring from a LN reared mother compared to offspring from SN and CN reared dams (Figure S2E), this was normalized at weaning at P21 (Figure S2F). Finally, basal levels of blood plasma corticosterone were not affected by rearing condition (Figure 4F).

### 3.3 Effects of heterozygous Drd4 knock‐out on F1

In males, but not females, heterozygous knock‐out of the dopamine receptor D4 (*Drd4* ^*+/−*^) resulted in a decreased body weight at weaning (Figure 2K). *Drd4* ^*+/−*^ mice did not differ from *Drd4* ^*+/+*^ animals in any of the sexual maturation measures (Figure 3). In addition, home‐cage maternal care levels towards the next generation were unaffected by *Drd4* genotype (Figure 4 and Figure S2). However, maternal responsiveness, as measured by pup retrieval, was improved in *Drd4* ^*+/−*^ dams compared to *Drd4* ^*+/+*^ animals (Figure S2D); *Drd4* ^*+/−*^ dams showed a higher completion rate in all rearing conditions (hazard ratio 95%CI = [1.03, 2.85], *P* = .040).

### 3.4 Moderation of rearing condition effects by Drd4 genotype

Different rearing conditions did not interact with *Drd4* genotype to determine body weight at weaning (Figure 2) or sexual maturation (Figure 3). In addition, basal corticosterone levels and most measures of maternal care were not affected by a gene‐early environment interaction (Figure 4). However, an interaction effect was observed for the percentage of time dams spent licking/grooming their own offspring (Figure 4C). In line with the differential susceptibility theory, *Drd4* ^*+/−*^ dams reared in the LN environment exhibited the lowest LG levels, whereas CN reared *Drd4* ^*+/−*^ mice spent the most time licking/grooming their own pups.

## 4 DISCUSSION

In this study, we examined the causal role of *Drd4* in differential susceptibility to the environment using a randomized experiment in rodents, allowing strict control for both genetic variation‐using *Drd4* ^*+/−*^ mice‐ and early‐life environmental factors. After extensive characterization of the effects of different environmental conditions on maternal care, we observed a differential susceptibility effect only for licking/grooming levels of adult female offspring towards their own litter. LN and CN reared *Drd4* ^*+/−*^ mice exhibited the lowest and highest levels of licking/grooming, respectively. In addition, we demonstrated main effects of rearing conditions on sexual maturation and maternal care towards the next generation. Mice reared in a limited nesting/bedding environment displayed characteristics of poor mothering, whereas communal nesting during early development resulted in higher predictability of maternal care.

### 4.1 Modeling impoverished and enriched rearing environments

The pattern of F0 maternal care resulting from exposure to the LN condition was largely in line with earlier findings using this model.^16^, ^17^, ^18^, ^19^ While different pup‐directed maternal behaviors remained relatively unaltered, the unpredictability of maternal behavior, particularly on the nest site, increased. In addition, pups in the LN condition were deprived from normal levels of licking/grooming upon first exposure to this condition on P2, whereas LG levels were similar to the SN and CN conditions from P3‐P8. In contrast to other reports, but in line with previous findings from our lab,^18^ fragmentation of maternal care was similar to control conditions, a difference that could be due to the difference in timing of observations. In this study, maternal behaviour was observed predominantly during the dark phase of the animals, whereas previous studies focused more on the light phase of the day/night cycle.^16^, ^19^ This difference in timing of observations is important as we observed, in line with earlier reports from our lab,^18^ a different circadian pattern in nest occupancy and ABN. LN dams exhibited altered circadian rhythmicity in maternal care, stressing the point that multiple time‐points or continuous monitoring across the day‐night should be examined to better grasp the implications of the LN condition.

Individual mouse dams adapted their maternal care to the communal nesting condition by decreasing nursing levels and increasing feeding behavior. Despite decreased nursing time per dam, offspring body weight was similar compared to SN reared animals. This could be explained in part by the observation that pups in the communal nesting condition have increased accessibility to at least one mouse dam, a hallmark of the early social enrichment provided by this model.^24^ In addition, litters in the CN condition are of a larger litter size, likely requiring less energy per pup to regulate body temperature.

### 4.2 Rearing conditions affect sexual maturation

The delayed puberty onset observed in both male and female LN reared mice was mediated by a decrease in body weight gain at weaning. The importance of body weight and leptin in regulating puberty onset is well‐known for both humans^40^, ^41^ and rodents.^42^ We therefore also measured body weight at puberty onset for the adolescent mice that were raised in different early life conditions. The minimal differences in body weight at puberty onset suggest that, irrespective of early life background and subsequent body weight at weaning, the majority of mice postpone the onset of puberty until a certain body weight is reached. This is in contrast to a recent study where body weight at vaginal opening was increased in female mice that were reared in a LN condition from P2‐9.^28^ However, because body weight at weaning of control groups is similar in both studies, this is unlikely to be a result of measurement differences. Future studies should therefore help to elucidate whether body weight at puberty onset is consistently affected by limited nesting rearing conditions.

In our study, only female mice reared in a CN setting showed increased body weight at puberty onset, indicating that these animals might exhibit, in line with the acceleration hypothesis, a *relative* delay in puberty onset, irrespective of bodyweight. It should be noted that early‐life adversity not only affects body weight but also alters adipose tissue, plasma leptin and leptin mRNA levels.^43^ Therefore, the mediation of puberty onset following LN is more complex and should be studied in more detail than only examining body weight per se. Nevertheless, the lack of differences in body weight at puberty onset between LN and SN reared mice, in combination with the delayed puberty onset of female mice that experienced increased unpredictability during rearing are not in line with the acceleration hypothesis of life history earlier proposed in humans. This may point to species differences but could also signify the relevance of uncontrolled factors in humans (eg, caloric intake) that are controlled for in the current design.

### 4.3 Rearing conditions affect later‐life maternal care

Different rearing conditions have been shown to affect maternal care provided to the next generation in the LN^32^ and CN^33^ models. Although previous results from our lab showed no effects of either LN or CN from P2‐9 on adult maternal behavior,^18^ the results presented here do support long‐lasting effects of rearing condition on maternal care. This could be explained by the duration and timing of exposure to early‐life rearing conditions (P2‐P9 in previous study compared to P2‐14 in this study). Given the different trajectories in brain circuit development,^44^, ^45^ the effects of early‐life adversity, and potentially also enrichment, strongly depend on the critical period during which it occurs.^46^ The importance of this critical or sensitive period is highlighted by a recent study showing that different windows of exposure to a combination of maternal separation with limited nesting differentially alter susceptibility to social defeat stress during adulthood.^47^ By extending the exposure of pups to different rearing conditions the development of brain regions involved in the regulation of maternal care, such as the MPOA and mPFC,^48^ may have been targeted more profoundly.

Extensive research from Meaney and co‐workers have identified the pivotal beneficial role of arched‐back nursing and licking/grooming behavior in rodent development.^31^, ^49^, ^50^ Many studies investigating intergenerational transmission of maternal care observe a similar phenotype in the offspring and the mother.^51^, ^52^ Interestingly, the lower ABN and nest occupancy levels of LN reared female mice observed in our current study did not coincide with a lower ABN or nest presence of their own mother. On the contrary, female LN‐reared pups experienced *increased* levels of nest occupancy by the dam compared to the SN condition, but showed *lower* levels of nest occupancy when taking care of a litter themselves. Similarly, CN reared mice received comparable levels of unpredictability as standard reared mice, yet provided more predictable maternal behavior towards their own offspring. Finally, LN‐reared animals received increased on‐nest unpredictability but showed similar on‐nest entropy rates compared to SN reared dams. Thus, although the differences in maternal care of F1 dams presented here are not mimicking the phenotype of the mother, the quality of the early‐life environment (poor vs enriched) did affect the quality of F1 maternal care under standard breeding conditions.

### 4.4 Drd4 genotype moderates the effects of rearing conditions

For licking/grooming behavior, the effects of rearing conditions were restricted to *Drd4* ^*+/−*^ animals, whereas rearing conditions had no effect on LG levels in wild‐type animals. Using *Drd4* genotype as a susceptibility factor, this is supportive evidence for differential susceptibility in a controlled animal model. Interestingly, the alterations were observed across generations, a finding that requires significant effort to study in humans. Studies on differential susceptibility in humans focused predominantly on the effects of maternal care on child development, highlighting the increased susceptibility of *DRD4‐7R* carrying children to parental sensitivity.^53^ However, as these studies have not yet examined parental care of the next generation, the translational relevance of results presented here is yet to be studied.

Clearly, the exact mechanisms through which the early‐life environment impacts on later‐life behavior remain to be elucidated. Previous studies suggest an important role for the methylation of genes involved in the HPA‐axis.^54^ Human studies also link the *DRD4‐7R* genotype to alterations in components of the HPA‐axis. Gene‐early environment effects have been observed for basal cortisol in children,^53^ as well as stress induced cortisol levels of young adults.^55^ A prominent role for alterations in circulating basal corticosterone levels in adulthood is not supported by our data. However, stress reactivity was not assessed and could, at least in part, underlie the observed alterations in maternal care.

Other systems may also be critical in the mechanism underlying differential susceptibility. Recent studies using different molecular tools and mouse knock‐in models have begun to unravel the exact function of the *DRD4‐7R* in corticostriatal glutamatergic neurotransmission, enhancing our understanding of the *Drd4* receptor and susceptibility to the environment.^56^, ^57^ Other studies used a wide array of techniques to show the involvement of other dopamine receptors in mediating the social deficits observed after severe early‐life stress.^58^ At a meta‐analytic level, however, the effects of early‐life adversity on the dopaminergic system appear limited, although significant for some parameters and areas.^59^ It is important to note that none of the studies included in the meta‐analysis examined *Drd4* as a potential target, highlighting the lack of preclinical evidence on the role of *Drd4* expression in mediating effects of adverse rearing conditions. The advances in our understanding of *Drd4* functioning at a molecular level and the role of other dopamine receptors in regulating susceptibility will help to guide future studies into the role of *DRD4*.

Finally, there is increasing awareness that most consequences of early‐life rodent models have small effect sizes,^21^ which is also the case in our study. Although we have sizable group numbers compared to common practice in the field, we should take this into consideration and interpret the results with care. To increase statistical power in future experiments, animal numbers should be adapted to realistically expected effect sizes and animal ethical committees should be aware of this.^60^ Moreover, more meta‐analyses in this field should be stimulated and can help in designing future studies.^21^

## 5 CONCLUSION

The research presented here provides a translational approach to examine the contribution of the *Drd4* gene in differential susceptibility. While other preclinical studies on differential susceptibility in socially monogamous prairie voles focused on the role of *prenatal* stress in enhancing developmental plasticity to both adverse and supportive contexts,^61^, ^62^ we show that adverse or enriched *postnatal* environments also interact with *genetic* factors in mice, for better and for worse. Future experiments should be targeted to test which neurobiological mechanisms are involved in mediating the effects of *DRD4* with regard to differential susceptibility.

## Supporting information


**Figure S1** Effect of different housing conditions on maternal care. (A) Feeding, (B) self‐grooming on nest, (C) self‐grooming off nest, (D) nest building, (E) other on nest and (F) other off nest behavior for limited nesting (red, n = 13), standard nesting (yellow, n = 14) and communal nesting (blue, n = 13) dams, depicted over postnatal days (left) and time of the day (right). The shaded area indicates the dark phase of the LD cycle. anova main effects are depicted in the top left of each figure. Post‐hoc comparisons are depicted bottom right or by lines. COND = main effect of condition. COND*OBS = condition*observation interaction effect. COND × PND = condition × postnatal day interaction effect. *P < 0.05, **P < .01, ***P < .001.
**Figure S2** Effects of different rearing conditions and *Drd4* genotype on F1 maternal care and F2 body weight. (A) Passive nursing and (B) total nursing levels. C, Total unpredictability rates. D, Pup retrieval latencies and completion rates. E, F2 offspring body weight at P2 and (F) P21. +/+: control, +/−: heterozygous *Drd4*. Group size: LN +/+: n = 10, LN +/−: n = 12, SN +/+: n = 16, SN +/−: n = 16, CN +/+: n = 10, CN +/−: n = 11). Asterisks indicate post hoc comparisons. *P < .05, **P < .01.
**Table S1** Number of animals per litter used in this study. Numbers in parentheses indicate the number of females that successfully raised a litter and were used for F1 maternal care. . +/+: control, +/−: heterozygous *Drd4*.
**Table S2** Statistical tests on the effects of different environmental conditions on F0 maternal care. P‐values in bold are considered statistically significant. P2 = postnatal day 2. P3‐8 = postnatal day 3‐8. *η* ^2^ = eta squared effect size.
**Table S3** Statistical tests on the effects of different rearing conditions on F1 outcome measures. *P*‐values in bold are considered statistically significant. P = postnatal day.

## Data Availability

The data that support the findings of this study are openly available through: https://osf.io/xw983/?view_only=315da0ae8cce430990777571abc5ab70
