# Supplementary material for: Maternal care of heterozygous dopamine receptor D4 knockout mice: Differential susceptibility to early‐life rearing conditions
Source: Genes Brain Behav. 2020 Jun 15;19(7):e12655. doi: 10.1111/gbb.12655 (PMC7540036; doi:10.1111/gbb.12655)
Supplement: Supplementary file 1 — Figure S1 Effect of different housing conditions on maternal care. (A) Feeding, (B) self‐grooming on nest, (C) self‐grooming off nest, (D) nest building, (E) other on nest and (F) other off nest behavior for limited nesting (red, n = 13), standard nesting (yellow, n = 14) and communal nesting (blue, n = 13) dams, depicted over postnatal days (left) and time of the day (right). The shaded area indicates the dark phase of the LD cycle. anova main effects are depicted in the top left of each figure. Post‐hoc comparisons are depicted bottom right or by lines. COND = main effect of condition. COND*OBS = condition*observation interaction effect. COND × PND = condition × postnatal day interaction effect. *P < 0.05, **P < .01, ***P < .001. Figure S2 Effects of different rearing conditions and Drd4 genotype on F1 maternal care and F2 body weight. (A) Passive nursing and (B) total nursing levels. C, Total unpredictability rates. D, Pup retrieval latencies and completion rates. E, F2 offspring body weight at P2 and (F) P21. +/+: control, +/−: heterozygous Drd4. Group size: LN +/+: n = 10, LN +/−: n = 12, SN +/+: n = 16, SN +/−: n = 16, CN +/+: n = 10, CN +/−: n = 11). Asterisks indicate post hoc comparisons. *P < .05, **P < .01. Table S1 Number of animals per litter used in this study. Numbers in parentheses indicate the number of females that successfully raised a litter and were used for F1 maternal care. . +/+: control, +/−: heterozygous Drd4. Table S2 Statistical tests on the effects of different environmental conditions on F0 maternal care. P‐values in bold are considered statistically significant. P2 = postnatal day 2. P3‐8 = postnatal day 3‐8. η 2 = eta squared effect size. Table S3 Statistical tests on the effects of different rearing conditions on F1 outcome measures. P‐values in bold are considered statistically significant. P = postnatal day. [file GBB-19-e12655-s001.pdf]

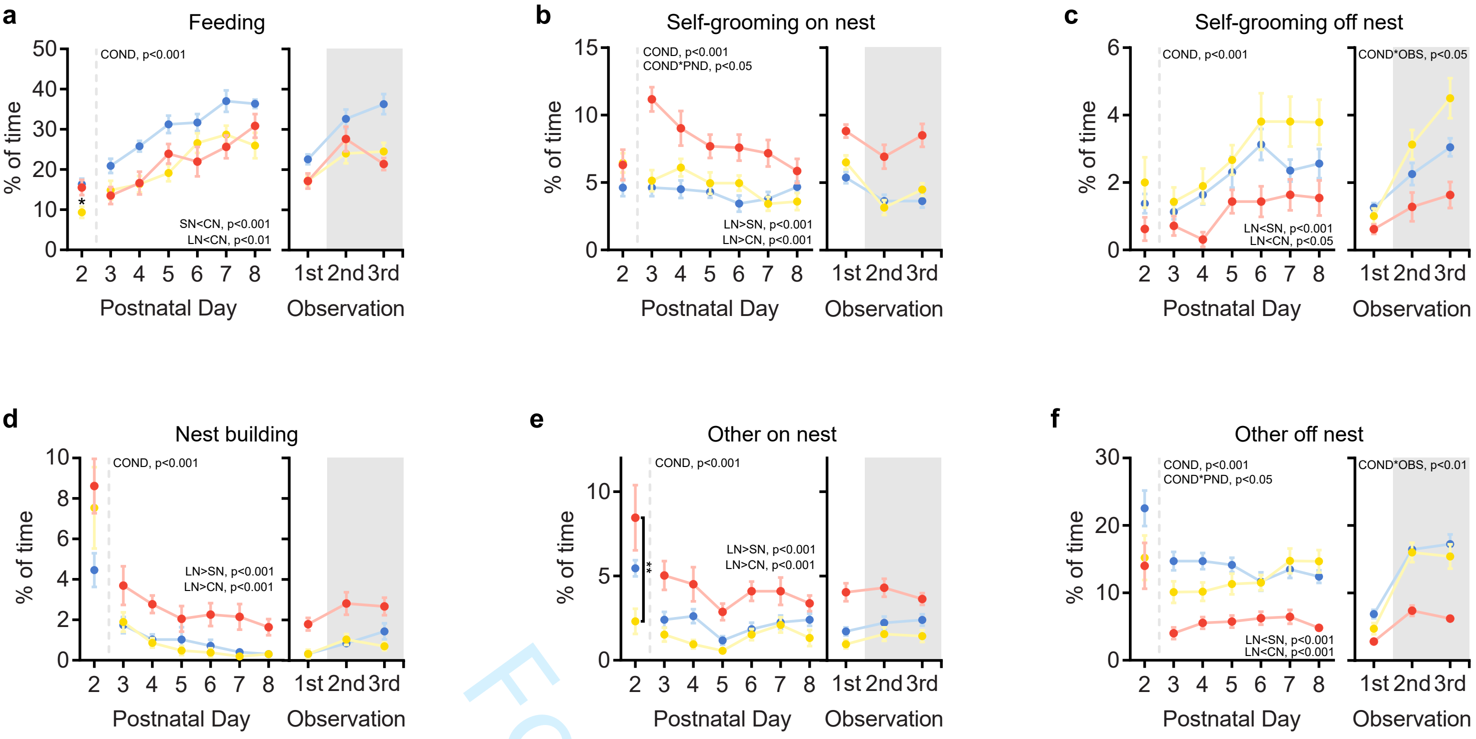

Figure S1 Effect of different housing conditions on maternal care.
(**a**) Feeding, (**b**) self-grooming on nest, (**c**) self-grooming off nest, (**d**) nest building, (**e**) other on nest and (**f**) other off nest behavior for limited nesting (red, n = 13), standard nesting (yellow, n = 14) and communal nesting (blue, n = 13) dams, depicted over postnatal days (left) and time of the day (right). The shaded area indicates the dark phase of the LD cycle. ANOVA main effects are depicted in the top left of each figure. Post-hoc comparisons are depicted bottom right or by lines. COND = main effect of condition. COND*OBS = condition*observation interaction effect. COND*PND = condition*postnatal day interaction effect. *p < 0.05, **p < 0.01, ***p < 0.001.

For Review Only

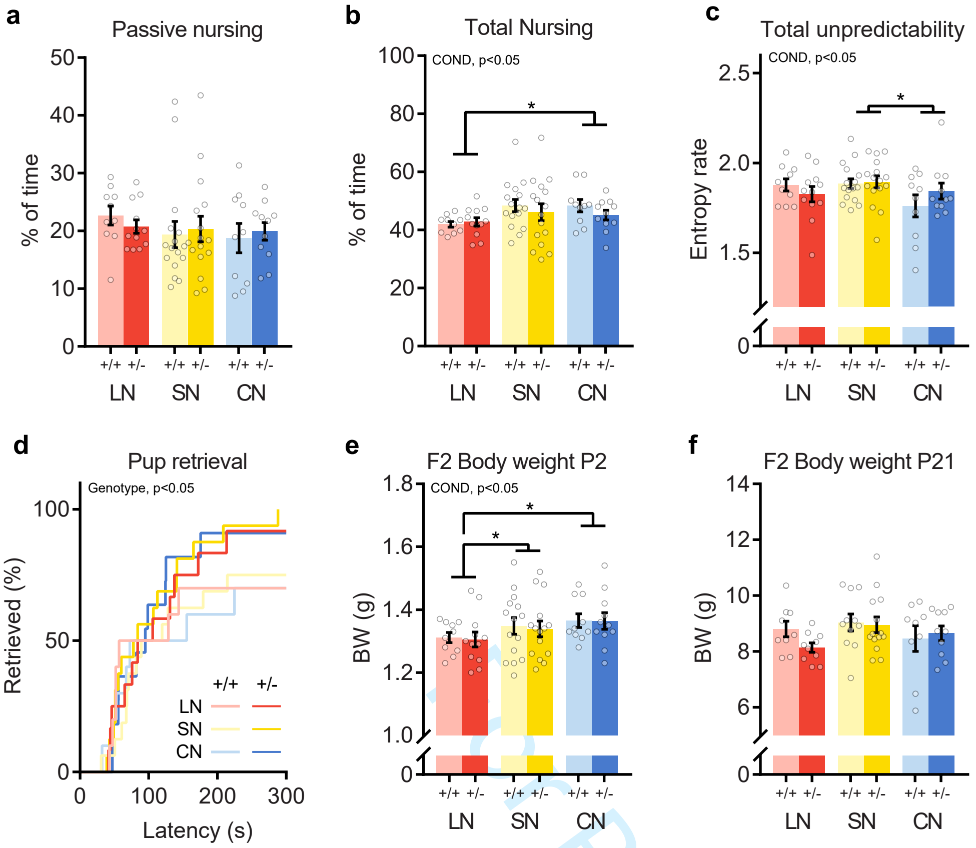

Figure S2 Effects of different rearing conditions and *Drd4* genotype on F1 maternal care and F2 body weight.

(**a**) Passive nursing and (**b**) total nursing levels. (**c**) Total unpredictability rates. (**d**) Pup retrieval latencies and completion rates. (**e**) F2 offspring body weight at P2 and (**f**) P21. +/+: control, +/-: heterozygous *Drd4*. Group size: LN +/+: n = 10, LN +/-: n = 12, SN +/+: n = 16, SN +/-: n = 16, CN +/+: n = 10, CN +/-: n = 11). Asterisks indicate post hoc comparisons. *$p < 0.05$, **$p < 0.01$.

For Review Only

	Litter	Males +/+	Males +/-	Females (mothers) +/+	Females (mothers) +/-
Limited Nesting	1	0	3	0 (0)	1 (1)
	2	1	1	3 (2)	1 (0)
	3	2	1	2 (1)	2 (1)
	4	2	1	2 (0)	2 (1)
	5	1	1	2 (0)	2 (1)
	6	1	2	1 (1)	3 (2)
	7	1	1	1 (1)	1 (1)
	8	1	3	1 (1)	2 (1)
	9	2	0	2 (1)	1 (1)
	10	2	0	4 (1)	0 (0)
	11	2	1	2 (2)	1 (1)
	12	1	2	2 (0)	2 (1)
	13	3	1	2 (0)	1 (1)
Standard Nesting	1	1	0	1 (1)	4 (3)
	2	1	3	0 (0)	1 (0)
	3	3	0	2 (1)	2 (2)
	4	1	3	2 (0)	1 (1)
	5	1	3	0 (0)	1 (0)
	6	1	2	3 (3)	0 (0)
	7	0	2	1 (0)	3 (3)
	8	0	0	1 (1)	4 (3)
	9	1	3	3 (2)	0 (0)
	10	2	1	1 (1)	1 (1)
	11	1	0	2 (1)	4 (2)
	12	0	2	4 (1)	0 (0)
	13	1	1	3 (2)	1 (1)
	14	0	3	3 (3)	0 (0)
Communal Nesting	1	0	3	2 (1)	1 (1)
	2	2	0	2 (2)	3 (1)
	3	2	2	1 (1)	1 (0)
	4	2	2	2 (1)	1 (0)
	5	2	1	1 (1)	3 (3)
	6	1	3	3 (1)	0 (0)
	7	2	3	0 (0)	3 (3)
	8	1	3	0 (0)	3 (2)
	9	1	3	1 (1)	2 (0)
	10	3	2	1 (0)	0 (0)
	11	3	1	2 (0)	0 (0)
	12	1	2	3 (2)	1 (1)
	13	2	2	2 (0)	0 (0)

Table S1 Number of animals per litter used in this study. Numbers in parentheses indicate the number of females that succesfully raised a litter themselves. +/+: control, +/-: heterozygous *Drd4*.

For Review Only

	P2				P3-8				Observation*condition			
	F-statistic	p-value	η^2	Post-hoc	F-statistic	p-value	η^2	Post-hoc	F-statistic	p-value	η^2	Figure
Individual behaviors												
Arched-back nursing	F(2, 36) = 2.92	.067	.14		F(2, 36) = 5.69	**.007**	.24	LN>CN	F(3.69, 68.31) = 3.40	**.016**	.07	2a
Passive nursing	F(2, 36) = 2.32	.112	.11		F(2, 36) = 5.10	**.011**	.22	SN>CN	F(3.93, 41.20) = 0.97	.428	.04	2b
Total nursing	F(2, 36) = 1.58	.219	.08		F(2, 36) = 14.27	**<.001**	.44	LN=SN>CN	F(3.75, 69.33) = 2.28	.073	.05	2c
Licking/grooming	F(2, 36) = 4.32	**.021**	.19	LN<SN=CN	F(2, 36) = 0.13	.880	.01		F(3.98, 73.70) = 0.80	.528	.04	2d
Time on nest	F(2, 36) = 3.00	.062	.14		F(2, 36) = 25.95	**<.001**	.59	LN>SN>CN	F(3.51, 64.86) = 2.72	**.044**	.06	2e
Feeding	F(2, 36) = 6.17	**.005**	.26	LN>SN<CN	F(2, 36) = 12.36	**<.001**	.41	LN=SN<CN	F(3.65, 67.43) = 2.31	.073	.07	S1a
Self-grooming on nest	F(2, 36) = 1.44	.250	.07		F(2, 36) = 19.59	**<.001**	.52	LN>SN=CN	F(3.98, 73.68) = 2.00	.104	.06	S1b
Self-grooming off nest	F(2, 36) = 1.88	.168	.09		F(2, 36) = 13.89	**<.001**	.44	LN<SN=CN	F(3.88, 71.73) = 3.60	**.011**	.10	S1c
Nest building	F(2, 36) = 2.12	.135	.11		F(2, 36) = 21.10	**<.001**	.54	LN>SN=CN	F(3.10, 57.35) = 0.78	.512	.03	S1d
Other on nest	F(2, 36) = 6.27	**.005**	.26	LN>SN	F(2, 36) = 28.83	**<.001**	.62	LN>SN=CN	F(3.83, 70.90) = 0.69	.594	.03	S1e
Other off nest	F(2, 36) = 2.16	.130	.11		F(2, 36) = 52.59	**<.001**	.75	LN<SN=CN	F(3.13, 57.96) = 4.20	**.008**	.08	S1f
Communal nesting	T-statistic	p-value	Cohen's D									
Any dam on nest	t(24) = -1.92	.067	0.75		F(1, 24) = 69.53	**<.001**	.74		F(1.63, 40.67) = 0.38	.641	.01	2f
Unpredictability/fragmentation												
Overall unpredictability					F(2, 37) = 2.70	.081	.13					2g
On-nest unpredictability					F(2, 37) = 16.02	**<.001**	.46	LN>SN=CN				2h
Fragmentation					F(2, 37) = 13.08	**<.001**	.41	LN=SN<CN				2i

Table S2 Statistical tests on the effects of different environmental conditions on F0 maternal care. P-values in bold are considered statistically significant. P2 = postnatal day 2. P3-8 = postnatal day 3 till 8. η^2 = eta squared effect size.

	Condition			Genotype		Condition*Genotype		
	F-statistic	p-value	Post-hoc	F-statistic	p-value	F-statistic	p-value	Figure
Body weight								
P14								
Litters	F(2, 37) = 32.44	**<.001**	LN<SN=CN	unknown		unknown		2j
P21								
Males	F(2, 36) = 8.77	**.003**	LN<SN=CN	F(1, 38) = 4.97	**.032**	F(2, 36) = 0.39	.686	2k
Females	F(2, 37) = 20.60	**<.001**	LN<SN=CN	F(1, 38) = 0.45	.505	F(2, 37) = 0.15	.829	2l
Puberty onset								
Males								
Puberty onset	F(2, 36) = 10.33	**.001**	LN>SN=CN	F(1, 37) = 2.83	.101	F(2, 36) = 1.84	.121	3a
Body weight at puberty onset	F(2, 34) = 2.20	.382		F(1, 35) = 0.38	.544	F(2, 34) = 0.20	.777	3b
Females								
Puberty onset	F(2, 38) = 13.09	**.003**	LN>SN=CN	F(1, 39) = 0.52	.477	F(2, 38) = 0.28	.947	3f
Body weight at puberty onset	F(2, 38) = 4.29	**.010**	LN=SN<CN	F(1, 39) = 0.09	.767	F(2, 38) = 0.06	.930	3g
F1 maternal care								
Maternal behavior								
Arched-back nursing	F(2, 33) = 4.02	**.027**	LN<SN	F(1, 34) = 3.01	.092	F(2, 33) = 1.32	.281	4a
Passive nursing	F(2, 33) = 0.73	.475		F(1, 34) = 0.00	.958	F(2, 33) = 0.58	.630	S2a
Total nursing	F(2, 33) = 4.79	**.024**	LN<CN	F(1, 34) = 1.14	.293	F(2, 33) = 0.94	.366	S2b
Licking/grooming	F(2, 33) = 4.51	**.011**	LN<CN	F(1, 34) = 0.10	.758	F(2, 33) = 4.99	**.028**	4c
Time on nest	F(2, 33) = 7.40	**.002**	LN<SN=CN	F(1, 34) = 0.33	.571	F(2, 33) = 0.13	.845	4b
Feeding	F(2, 33) = 1.70	.165		F(1, 34) = 3.31	.078	F(2, 33) = 0.23	.845	-
Self-grooming on nest	F(2, 33) = 2.02	.146		F(1, 34) = 3.37	.075	F(2, 33) = 0.90	.331	-
Self-grooming off nest	F(2, 33) = 0.24	.763		F(1, 34) = 0.72	.401	F(2, 33) = 0.92	.424	-
Nest building	F(2, 33) = 1.03	.350		F(1, 34) = 2.13	.153	F(2, 33) = 1.09	.453	-
Other on nest	F(2, 33) = 0.42	.806		F(1, 34) = 1.53	.224	F(2, 33) = 2.52	.058	-
Other off nest	F(2, 33) = 2.88	.055		F(1, 34) = 0.47	.498	F(2, 33) = 0.11	.893	-
Unpredictability/fragmentation								
Total entropy	F(2, 33) = 3.20	**.032**	SN>CN	F(1, 34) = 0.12	.733	F(2, 33) = 0.72	.411	S2c
On-nest entropy	F(2, 33) = 3.62	**.044**	LN>CN	F(1, 34) = 0.31	.579	F(2, 33) = 0.85	.374	4d
Fragmentation	F(2, 33) = 1.08	.269		F(1, 34) = 0.12	.728	F(2, 33) = 0.55	.505	4e
F2 body weight								
P2	F(2, 33) = 5.48	**.012**	LN<SN=CN	F(1, 34) = 0.11	.746	F(2, 33) = 0.03	.964	S2e
P21	F(2, 30) = 2.36	.566	LN<SN=CN	F(1, 31) = 1.56	.221	F(2, 30) = 0.69	.630	S2f
Corticosterone	F(2, 32) = 0.10	.904		F(1, 33) = 2.04	.163	F(2, 32) = 0.55	.532	4f

Table S2 Statistical tests on the effects of different rearing conditions on F1 outcome measures. P-values in bold are considered statistically significant. P = postnatal day.
